# Seeing the talker’s face supports executive processing of speech in steady state noise

**DOI:** 10.3389/fnsys.2013.00096

**Published:** 2013-11-26

**Authors:** Sushmit Mishra, Thomas Lunner, Stefan Stenfelt, Jerker Rönnberg, Mary Rudner

**Affiliations:** ^1^Linnaeus Centre HEAD, Swedish Institute for Disability Research, Department of Behavioural Sciences and Learning, Linköping UniversityLinköping, Sweden; ^2^Department of Clinical and Experimental Medicine, Linköping UniversityLinköping, Sweden; ^3^Eriksholm Research Centre, Oticon A/SSnekkersten, Denmark

**Keywords:** cognitive spare capacity, executive processing, working memory, updating, inhibition, speech processing

## Abstract

Listening to speech in noise depletes cognitive resources, affecting speech processing. The present study investigated how remaining resources or cognitive spare capacity (CSC) can be deployed by young adults with normal hearing. We administered a test of CSC (CSCT; Mishra et al., 2013) along with a battery of established cognitive tests to 20 participants with normal hearing. In the CSCT, lists of two-digit numbers were presented with and without visual cues in quiet, as well as in steady-state and speech-like noise at a high intelligibility level. In low load conditions, two numbers were recalled according to instructions inducing executive processing (updating, inhibition) and in high load conditions the participants were additionally instructed to recall one extra number, which was the always the first item in the list. In line with previous findings, results showed that CSC was sensitive to memory load and executive function but generally not related to working memory capacity (WMC). Furthermore, CSCT scores in quiet were lowered by visual cues, probably due to distraction. In steady-state noise, the presence of visual cues improved CSCT scores, probably by enabling better encoding. Contrary to our expectation, CSCT performance was disrupted more in steady-state than speech-like noise, although only without visual cues, possibly because selective attention could be used to ignore the speech-like background and provide an enriched representation of target items in working memory similar to that obtained in quiet. This interpretation is supported by a consistent association between CSCT scores and updating skills.

## Introduction

Listening in noise challenges explicit cognitive abilities (Rönnberg et al., 2008, 2013). A substantial body of work has shown that once audibility has been accounted for, individual working memory capacity (WMC) accounts for a large part of the variance in the ability to understand speech in noise (Humes, 2007; Akeroyd, 2008). This is not surprising as speech understanding requires encoding of the speech input for temporary storage, inferring meaning and at the same preparing for an appropriate response (Pichora-Fuller and Singh, 2006; Rudner and Lunner, 2013). Because WMC is limited, fewer cognitive resources will remain after processing of a message heard in noise compared to one heard in quiet (Pichora-Fuller, 2007; Lunner et al., 2009). This line of argument has sparked recent interest in measuring cognitive spare capacity (CSC), that is, the cognitive capacity that remains once successful listening has taken place (Rudner et al., 2011a). The concept of CSC is similar to the concept of WMC. However, whereas WMC often refers to a general capacity that is typically measured using a text-based visual task such as the reading span task (Daneman and Carpenter, 1980; Rönnberg et al., 1989), CSC refers specifically to a cognitive reserve that has been depleted by listening under adverse conditions such as against a background of noise or with a hearing impairment. It is likely that different cognitive functions do not tap into a single cognitive resource but have their own dedicated and distinct resources (Mishra et al., 2013). During speech understanding, a cognitive resource that is depleted in the act of speech perception may be compensated for fully or partially by another cognitive process. Hence, a measure of WMC may not provide adequate assessment of CSC. Being able to gauge individual CSC may be an important factor in designing and evaluating interventions for individuals with various communication difficulties. For example, it is likely to assist in appropriate fitting of hearing aids (Rudner et al., 2011a; Mishra et al., 2013).

In a recent publication (Mishra et al., 2013), we evaluated a test of CSC (CSCT) that probes the ability to perform different executive tasks (updating and inhibition) under high and low memory load based on two-digit numbers presented in the auditory modality, with or without a video of the talker’s face. We found that CSC, measured using the CSCT, did not correlate with WMC, measured using the reading span task, suggesting that CSC is quantitatively and qualitatively different from WMC. Rather surprisingly, we found that CSC was reduced when the speaker’s face was visible when the to-be-remembered stimuli were presented in quiet conditions. We suggested that when speech is fully audible and there is no competing noise, seeing the talker’s face may act as a distraction during performance of the executive tasks. This interpretation is in line with other recent work demonstrating that when the intelligibility of audiovisual (AV) stimuli is equated with that of auditory-only (A-only) stimuli, listening effort gauged using a dual task procedure increases in the AV modality (Fraser et al., 2010; Gosselin and Gagné, 2011). The purpose of the present study was to replicate the findings of Mishra et al. (2013) and to investigate CSC for speech presented in noise.

The Ease of Language Understanding model (ELU; Rönnberg, 2003; Rönnberg et al., 2008, 2013) assumes that in optimal listening conditions, the incoming language signal can be readily matched with lexical and phonological representations stored in Long Term Memory (LTM), making speech understanding implicit. But in the presence of adverse conditions which may include noise or signal degradation (Mattys et al., 2012), a mismatch may occur between the incoming signal and stored representations. In a mismatch situation, explicit or effortful conscious processing is required to infer meaning from the incoming fragments of information. Such processing may include the abilities to achieve linguistic closure and gain access to previous knowledge stored in LTM (Rönnberg et al., 2010; Besser et al., 2013). Also, it has been suggested that individuals may compensate for information lost during signal degradation by directing their attentional capacity towards understanding the signal (Mattys et al., 2012). The use of explicit processing or involvement of attentional capacity for speech perception involves the executive functions of updating and inhibition (Mishra et al., 2010, 2013; Rudner et al., 2011a; Sörqvist and Rönnberg, 2012; Rönnberg et al., 2013). Updating refers to the monitoring and coding of information which is relevant to the task at hand and inhibition involves deliberate, controlled suppression of prepotent responses (Miyake et al., 2000). For example, while listening in modulated noise, the executive function of inhibition is used to suppress distracting noise (Janse, 2012). The involvement of executive functions in the act of listening, especially under adverse listening conditions, may come at the cost of depleted cognitive resources for processing heard material. This means that modulated noise may be more disruptive of performance on the inhibition than the updating task of the CSCT and that individual executive ability may specifically predict the ability to process speech heard in noise (Rudner et al., 2011b).

The ability to perceive speech (Mattys et al., 2012) and to remember speech (Pichora-Fuller et al., 1995; Murphy et al., 2000; Sörqvist and Rönnberg, 2012) is influenced in different ways by different types of noise. It has been regularly observed that speech recognition scores are higher in modulated compared to steady state noise at similar signal-to-noise ratios (SNR) (e.g., Duquesnoy, 1983) at least for younger adults without hearing impairment (George et al., 2006; Zekveld et al., 2013). However, speech recognition in modulated compared to steady state noise is also more likely to be associated with individual cognitive abilities such as WMC (Rönnberg et al., 2010; Besser et al., 2013; Zekveld et al., 2013) and linguistic closure (Zekveld et al., 2007; Besser et al., 2013). It is also likely to be more effortful both in terms of physiological response such as pupil dilation (Koelewijn et al., 2012) and subjective self-ratings, even when performance is better (Rudner et al., 2012). Recently, Janse (2012) showed that listening to speech in speech noise demands inhibition. These findings suggest that better speech recognition in modulated compared to steady state noise is dependent on cognitive resources. This applies in particular to speech noise (Zekveld et al., 2013) and hence CSC may be reduced more by listening to speech in speech-like noise than in steady state noise. Noise has been shown to disrupt recall of spoken items even when intelligibility is fairly high (Murphy et al., 2000). Emerging evidence suggests that speech-like noise, compared to steady state noise, is more disruptive of short-term memory retention of fully audible items in older adults with hearing loss (Ng et al., 2013). Thus, in the present study, we expected lower CSCT scores in noise than in quiet, but higher in steady state than speech-like noise. Because speech recognition in speech-like noise seems to specifically tax inhibition resources, we speculated that listening in speech-like noise might actually reduce the inhibition resources available for performing the CSCT. Thus, we expected speech-like noise to lower CSCT scores more in the inhibition than updating task.

Speech perception in the presence of noise is usually enhanced by observation of the talker’s face. Lips, teeth and tongue may provide disambiguating information that is complementary to less well specified auditory information, by helping to determine manner and more importantly place of articulation (Grant et al., 1998). This can provide a substantial benefit in signal to noise ratio (Campbell, 2009) for speech recognition in noise (see also Hygge et al., 1992). The advantage of AV presentation has even been observed when only a graphic representation of the movement of the articulators was shown during speech detection in noise (Tye-Murray et al., 2011). Such visual cues do not provide disambiguating information and thus this finding was interpreted as suggesting that visual cues help the listener to direct their attentional capacities to the incoming signal at the most critical time to encode the target (c.f. Helfer and Freyman, 2005). This causes better signal detection and fewer cognitive demands in anticipating target stimuli in AV compared to A-only presentation (Besle et al., 2004; Moradi et al., 2013). Thus, visual cues help segregate target stimuli from interfering noise. Notwithstanding, it has been found that visual cues reduce performance on executively demanding auditory tasks (Fraser et al., 2010; Gosselin and Gagné, 2011; Mishra et al., 2013).

In a recent article, Yovel and Belin (2013) suggested that despite sensory differences, the neurocognitive mechanisms engaged by perceiving faces and voices are highly similar, facilitating integration of visual and speech information. Indeed, recent investigations of the episodic buffer of working memory (Baddeley, 2000) have shown that contrary to predictions, multimodal integration in working memory is not executively taxing (Rönnberg et al., 2010; Baddeley, 2012). One of the characteristics of the episodic buffer is that it communicates with LTM and exploits the quality of representations stored there (Rönnberg et al., 2013). Moradi et al. (2013) found that the AV speech recognition in the presence of noise for subjects with normal hearing is faster, more accurate and less effortful than A-only speech recognition and inferred that AV presentation taxes cognitive resources to a lesser extent by reducing working memory load. On the basis that AV presentation provides additional complimentary visual cues and helps in anticipating target onset in the presence of noise, we predict that seeing the face of the talker while performing the CSCT in noise will enable participants to form richer representations of the target items that will better survive the executive processing that the task demands. We do not expect visual information to increase the cognitive burden when CSCT is performed in noise. However, in our previous study, CSCT scores in quiet were found to be higher without visual cues in participants with normal hearing (Mishra et al., 2013). This is line with other work showing that visual cues may interfere with performance on executively challenging tasks (Fraser et al., 2010; Gosselin and Gagné, 2011) and may be due to difficulties in prioritizing task-related processing in the presence of low-priority stimuli (Lavie, 2005). In the quiet conditions of the CSCT, the numbers are fully audible and thus visual cues become low-priority stimuli. Thus, the superfluous visual information in the AV modality may act as a distractor when it is not required to segregate the target stimuli from a noise background. Hence, in the present study, we predicted higher CSCT scores in A-only compared to AV modality in quiet and the opposite in noise.

The main purpose of the present study was to investigate how noise influences CSC. In summary, we predicted that noise would disrupt executive processing of intelligible auditory two-digit numbers leading to lower CSCT performance than in quiet. Speech-like noise would be more disruptive than steady-state noise given the same SNR, particularly during the inhibition task. Seeing the talker’s face would counteract the noise decrement by helping the listener segregate target from noise and generate richer cognitive representations. However, in quiet conditions, visual cues would act as a distractor and reduce performance.

We have argued that cognitive skills like WMC (Pichora-Fuller and Singh, 2006; Rudner and Lunner, 2013; Rönnberg et al., 2013), executive functions (Mishra et al., 2013), linguistic closure and LTM (Rönnberg et al., 2010) are engaged during the complex processing involved in speech understanding. Moreover, linguistic closure and LTM may play a specific role in solving the updating and inhibition tasks in the CSCT. Even if only part of a two-digit number has been perceived it may provide task-relevant information. For example in an updating task requiring retention of the highest numbers by each speaker, a new list number in the twenties by a particular speaker can be discarded if the retained number by the same speaker is in the thirties or higher. In this particular case it is sufficient to achieve LTM access and linguistic closure for the part of the number that provides the necessary information. To assess the contribution of individual cognitive functions towards CSC and speech intelligibility, we administered a cognitive test battery in the present study along with the CSCT. This battery included, the reading span test (Daneman and Carpenter, 1980; Rönnberg et al., 1989) as a measure of WMC, the Text Reception Threshold test (TRT; Zekveld et al., 2007) as a measure of linguistic closure, the Letter Memory Task (Morris and Jones, 1990; Miyake et al., 2000) as a measure of updating, the Simon task (Simon, 1969; adapted from Pratte et al., 2010) as a measure of inhibition, and delayed recall of the reading span stimuli to measure episodic LTM. We did not predict an overall association between CSCT performance and reading span because reading span measures WMC and not specifically the ability to deploy executive processing resources during listening in noise. However, we did expect an association with TRT and delayed recall of the reading span stimuli because linguistic closure, which TRT measures, and LTM, which delayed recall measures may play key roles in CSC. We predicted that performance on the updating and inhibition tasks of the CSCT would be associated with the respective independent measures of executive function and that individual executive abilities would be more predictive of CSCT performance in noise than in quiet.

## Methods

### Participants

Twenty native Swedish speakers with either continuing or completed university education, including 11 females and 9 males, participated in the experiment. They were 19–35 years of age, M = 25.9; SD = 4.4. Hearing thresholds of the participants were within normal limits (better than 25 dB HL) in the frequency range of 125–8 kHz. The participants did not report any psychological or neurological problems. Visual acuity after correction was normal as measured by the Jaeger eye chart (Weatherly, 2002). Ethical approval for the study was obtained from the regional ethical review board, Linköping, Sweden.

### Material

The CSCT stimuli consisted of AV and A-only recordings of the Swedish two-digit numbers 13–99 spoken by a male and a female native Swedish speaker (Mishra et al., 2013). The levels of the numbers were equated for 50% intelligibility in steady state noise using a group of ten young adults with normal hearing. This was accomplished by increasing the SNR in steps of 1 dB for each number until a correct response was given in a procedure similar to that described in Hällgren et al. (2006). For more details of CSCT materials, see Mishra et al. (2013).

### Noise

The stationary noise was a steady-state speech-weighted (SSSW) noise, having the same long term average spectrum as the recorded numbers. The modulated noise was the International Speech Testing Signal (ISTS; Holube et al., 2010). The ISTS noise is designed to be speech-like but unintelligible and is thus composed of concatenated speech segments of around 10 ms duration in six languages (American English, Arabic, Mandarin, French, German and Spanish) spoken by six different female speakers. The stimuli and noises were calibrated for the same root mean square (RMS) levels initially and then the noise levels were changed keeping the speech level constant to obtain individualized SNRs.

### Individualizing SNR

The stimulus materials (numbers) in A-only modality and the SSSW noise were used in an adaptive procedure to determine the individualized SNR for presentation of the CSCT. In this adaptive procedure, the first stimulus was presented at an SNR of 5 dB and the participants were instructed to repeat the numbers they heard and were encouraged to guess if they were unsure. For the first run, for each new presented number that was repeated correctly, the noise was increased by steps of 3 dB until the participant’s response was incorrect. Thereafter, the step size was changed to 1 dB and 30 numbers were randomly selected and presented consecutively to determine the 84% intelligibility level adaptively in a four-down/one-up procedure (Levitt, 1971). In the second step, the SNR obtained for 84% intelligibility was increased by 0.5 dB to give an approximate intelligibility level of 90% in SSSW noise. This 0.5 dB increment yielded an approximate intelligibility level of 90% in SSSW noise as verified in a piloting study using six participants with normal hearing. The 90% level was chosen so that the participants could perceive most numbers, in order to perform the tasks in CSCT, but requiring effort. To verify the intelligibility at this new SNR, 60 numbers, again randomly selected from the stimulus material, were used. These numbers were presented at the set SNR and the intelligibility with SSSW and ISTS noise was obtained independently. The same individualized SNR levels were applied in the SSSW and ISTS noise during CSCT presentation. The above tests were implemented in MATLAB (Version 2009b).

## Cognitive spare capacity test (CSCT)

In the CSCT (Mishra et al., 2013), 48 lists of 13 two-digit numbers (13–99) are presented serially and after each list the participant is requested to report two specified list items, depending on the predetermined criteria. These were designed to elicit updating and inhibition. In the updating task, the participants are asked to recall either the highest (in one version) or the lowest (in the other version) value item spoken by the male and female speaker in the particular list. In the inhibition task, the participants are asked to recall either two odd (in one version) or even (in the other version) value items spoken by a particular speaker. In half the trials, two numbers only are reported; these are low memory load trials. These two numbers are never the first item in the list. In the other half, high memory load trials, the first number in the list is also reported along with the two specified items, i.e., three numbers in total need to be held in working memory but only two of them are subject to processing. The first number (dummy item) in the high memory load trials is not included in the scoring. Thus, all scoring in the CSCT is based on correct report, in any order, of two numbers. These tasks are performed with either AV or A-only stimulus presentation and in the present study presentation took place in quiet (no noise), SSSW noise and ISTS noise.

The CSCT was administered using DMDX software (Forster and Forster, 2003; Mishra et al., 2013). The participants performed all conditions of each of the executive tasks in a balanced order in a single block. Thus, over both task blocks, there were a total of 24 conditions of presentation in the CSCT with two executive tasks, two memory loads, two modalities of presentation and three noise conditions in a 2 × 2 × 2 × 3 design. Two lists per condition were tested. The order of the conditions was pseudo-randomized within the two executive task blocks and balanced across the participants. For the noisy conditions, the noise sound files were played together with the AV and A-only stimulus files in DMDX with noise onset 1 s prior to stimulus onset and offset of at least 1 s after stimulus offset. The lists of numbers were always presented at 65 dB SPL and the level of the noise was varied depending upon the individualized SNR level. Across all the conditions (noisy or quiet), the duration of presentation of each list was 33 s. The time from onset of one stimulus to the onset of the next was 2.5 s. The visual stimuli were presented using a computer with a screen size of 14.1 inches and screen resolution of 1366 × 768 pixels. The video was displayed in 720 × 576 pixels resolution in the center of the screen and the auditory stimuli were presented through Sennheiser HDA 200 headphones.

Before each of the executive task blocks, the participants were provided with written instructions for the particular executive task and the instructions were also elaborated verbally. Before each list, the participant was prompted on the computer screen as to which version of the executive task was to be performed, what the modality was and whether to remember two or three numbers (low or high load). This prompt remained on screen until the participant pressed a button to continue to the test. The order of the noise conditions was pseudo-randomized. At the end of each list, an instruction “Respond now” appeared on the screen and the participant was required to report the target numbers orally. The participants could make corrections in reported numbers and then pressed another button when they were ready to continue. The oral responses of the participants were audio recorded. The participants were specifically instructed to keep looking at the screen during stimulus presentation. This applied even during presentation in the A-only modality where a fixation-cross was provided at center screen. All the participants practiced each task with two lists before doing the actual test.

### Cognitive test battery

#### Reading span test

In the reading span test (Daneman and Carpenter, 1980; Rönnberg et al., 1989), the participant read series of sentences in Swedish consisting of three words which appeared on the computer screen one at a time. Each word was shown for 800 ms with an interval of 50 ms between words. Each series consisted of three to six sentences presented in increasing series length. Half of the sentences were coherent and half were nonsense. The participants’ task was to make a sematic judgment in 1.75 s and respond “yes” (if the sentence was coherent) or “no” (if the sentence was nonsense) before the next sentence appeared. At the end of each series of sentences, the participants were prompted by an instruction on the screen to recall either the first or the last word of all the sentences in the series in the order they appeared on the screen. The participants were provided with written instructions about the test and they did practice with a series of three sentences before the actual testing. There were a total of 54 sentences and the total score obtained on the recall in any order of first and final words was used in the analysis.

#### Text reception threshold (TRT)

The TRT (Zekveld et al., 2007) test is a visual analogy of a test of speech recognition in noise and measures ability to read partially masked text. A Swedish version of the TRT, using the Hearing In Noise Test (HINT) sentences (Hällgren et al., 2006) was used. The test consisted of presentation of three lists of 20 HINT sentences each, the first list being a practice list. The sentences appeared word by word on the screen in red masked by vertical black bars with the preceding words remained on the screen until the sentence was completed. After presentation of the last word, the sentence remained visible for 3.5 s. The presentation rate of the words in each sentence was equal to the speaking rate in the corresponding speaker file. A one-up-one-down adaptive procedure with a step-size of 6% was applied to target percentage of unmasked text required to read 50% of the sentences entirely correct. The average percentage of unmasked text from the two lists of sentences was used as dependent variable.

#### Letter memory task

In the letter memory task (Morris and Jones, 1990; Miyake et al., 2000), the participants were presented with sequences of letters and asked to hold the four most recent letters in mind and then prompted to say them at the end of each sequence. Responses were audio-recorded. DMDX software was used to present lists of 5, 7, 9 or 11 consonants serially at the center of the computer screen. Two lists consisting of 7 and 9 letters were presented as practice and the testing consisted of 12 lists. Sequence length of test lists was randomized across trials to ensure that the participant followed the instructed strategy and continuously updated working memory until the end of the trial. The score was the number of consonants correctly recalled irrespective of order.

#### Simon task

A visual analogue of the go/no go task (Simon, 1969; adapted from Pratte et al., 2010), using red and blue rectangular blocks which appeared on the left or the right of the computer screen successively at intervals of 2 s. The participants were required to respond as quickly as possible by pressing a button on the right hand side of the screen when they saw a red block and when they saw a blue block they pressed a button on the left hand side of the screen. A total of 16 blocks were presented using DMDX without a practice. The participant had to ignore the spatial position in which the block appeared in the task and when the spatial position of the stimulus and correct response key coincided, the trial was termed congruent otherwise incongruent. The difference in reaction time between the incongruent and congruent trials was taken as a measure of inhibition.

#### Delayed recall of reading span test

Delayed recall of the items presented during the reading span test was used to assess episodic LTM. In this test the participants were asked to recall, without forewarning, words or whole sentences remembered from the reading span test after approximately 60 min. During the 60 min, the participants performed the other tests in the cognitive test battery. The score on delayed recall test was the total number of words correctly recalled by the participant irrespective of the order.

### Procedure

The participants, on arriving for the testing, were fully briefed about the study and a consent form was signed. All the participants underwent vision screening and audiometric testing in the audiometric booth. The testing was conducted in two sessions. All auditory testing took place in a sound-treated booth with the participants facing the computer screen. Each session took approximately 90 min. The reading span test was administered followed by the Simon task, the letter memory test and the TRT test in a separate room. Individual SNRs for the CSCT were determined and the delayed recall of the reading span test concluded the first session. In the second session, the CSCT test was conducted. The participants were allowed to take breaks after different tests. Written instructions were provided for all the tests and the participants were given the opportunity to request oral clarification.

### Data analysis

A repeated measures analysis of variance (ANOVA) on the CSCT scores was conducted. In order to test our a-priori hypotheses, planned comparisons were carried out and the simple main effect observed without a-priori hypothesis was investigated using post-hoc Tukey’s Honestly Significant Difference (HSD) test. In order to assess the association between cognitive functions and speech intelligibility and CSCT, Pearson’s correlations with the cognitive test battery were computed.

## Results

### Intelligibility

In the noise conditions, the mean SNR for CSCT presentation was −2.17 dB (SD = 0.85). The mean intelligibility level was 93.8% (SD = 3.0) and 92.3% (SD = 2.9) for the SSSW and ISTS noise respectively. There was no statistically significant difference in speech intelligibility performance in SSSW and ISTS noise (*t* (38) = 1.58, *p* = 0.12).

### Cognitive spare capacity test (CSCT)

**Figure 1:**
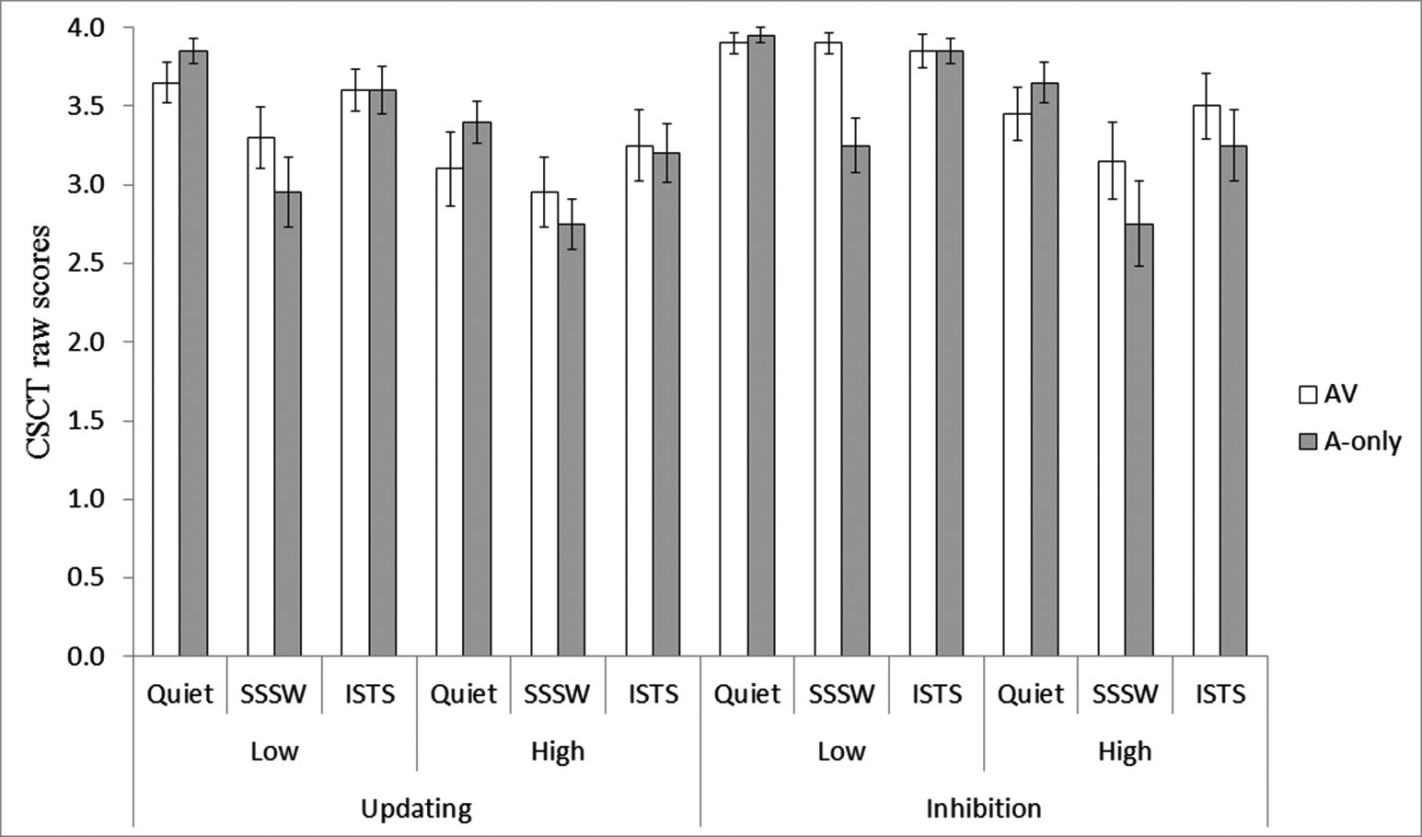
**Mean raw CSCT score for the AV (unfilled bars) and A-only (filled bars) modalities of presentation in the high and low memory load conditions of the updating and inhibition tasks in the three noise conditions**. Error bars represent standard errors.

Figure 1 displays the mean scores obtained in the 24 conditions of CSCT. Since there were two lists presented per condition, the maximum score per condition was four. Performance in the inhibition task in the low memory load conditions approached ceiling, and so all analyses of CSCT data were conducted on the rationalized arcsine-transformed scores (Studebaker, 1985) to counteract data skewing. Mean recall of the dummy item in the high memory load conditions, was 22.4 (SD = 1.3) out of 24 possible responses. This demonstrates that the participants carried out the CSCT task in the high memory load conditions according to the instruction.

The repeated measures ANOVA of the CSCT scores revealed main effects of all four factors: executive function, *F*(1, 19) = 10.01, MSE = 0.28, *p* < 0.01, memory load, *F*(1, 19) = 28.14, MSE = 0.23, *p* < 0.01, modality, *F*(1, 19) = 4.59, MSE = 0.10, *p* < 0.05 and noise, *F*(2, 38) = 18.25, MSE = 0.18, *p* < 0.01. The main effect of executive function and memory load showed higher CSCT scores in inhibition than updating conditions and in low than high memory load conditions, in line with the predictions. Also, CSCT scores were higher in AV than A-only conditions. In order to identify significant differences in performance between the three levels of noise, pair-wise comparisons with Bonferroni adjustment for multiple comparisons were conducted. They revealed that CSCT scores in quiet and ISTS noise was significantly higher than in SSSW noise (*p* < 0.05) but there was no significant difference between the performance in quiet and ISTS noise (*p* = 1.00), see Figure 1.

**Figure 2:**
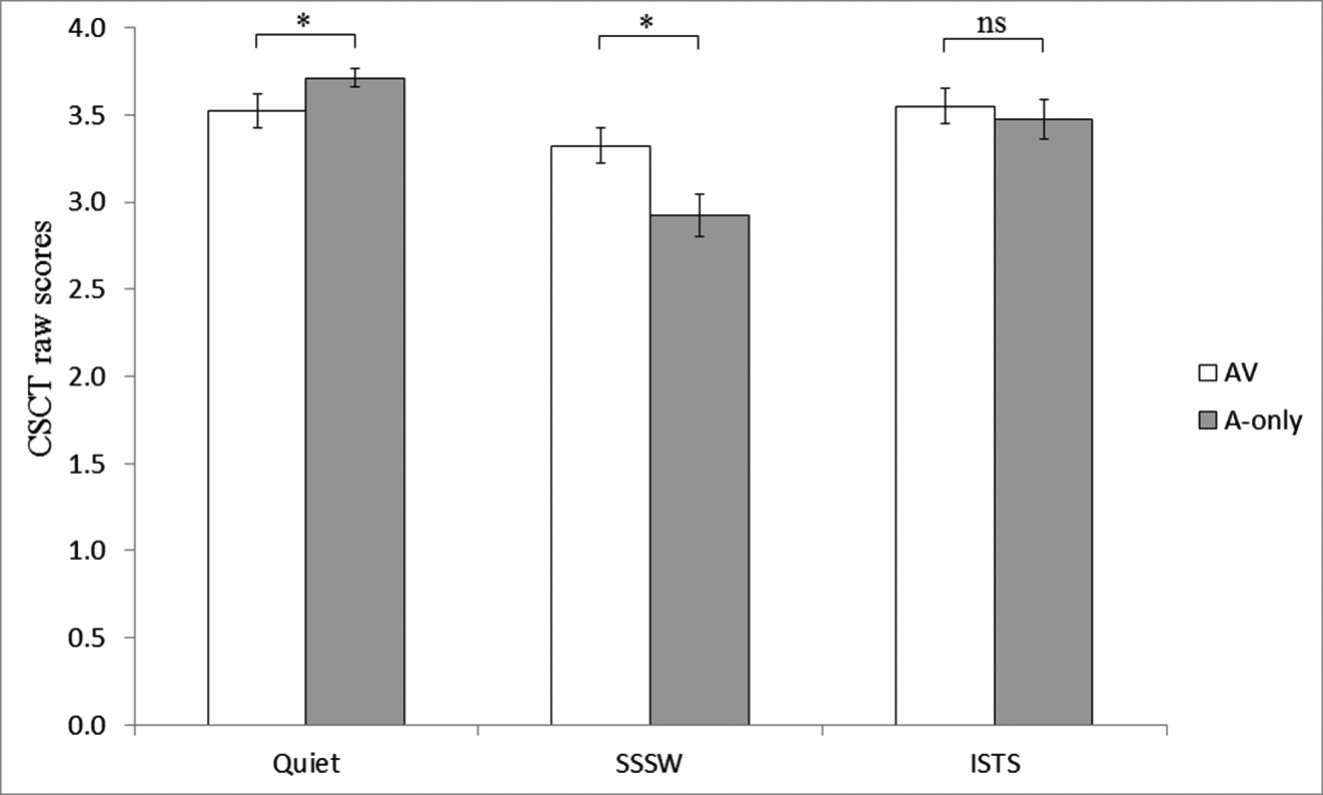
**Significant two-way interaction between modality of presentation and noise**. Raw scores for AV (unfilled bars) and the A-only modalities of presentation (filled bars) in the three noise conditions of CSCT are represented. Error bars represent standard errors. * Indicates significance at 0.05 level (1-tailed).

The two-way interaction between noise and modality was significant, *F*(2, 38) = 6.78, MSE = 0.14, *p* < 0.01. We had a hypothesis that performance would be better in the A-only than AV modality in quiet but the opposite in noise. Planned comparisons revealed better performance in A-only compared to AV (*t* = 1.86, *p* < 0.05, one tailed) in quiet, and better performance in AV compared to A-only (*t* = 2.52, *p* < 0.05, one tailed) in SSSW in line with our predictions, see Figure 2. However, in ISTS there was no significant difference between performance in AV and A-only conditions (*t* = 1.1, *p* > 0.05, one tailed).

Post-hoc Tukey’s HSD tests assessing the two-way interaction revealed that in the A-only modality of presentation, the scores was significantly higher in quiet (*p* < 0.01) and ISTS noise (*p* < 0.01) compared to SSSW noise and also that there was no significant difference between performance in AV modality in SSSW noise and A-only modality in ISTS noise, see Figure 2.

We had predicted that modulated noise would disrupt the inhibition task more than the updating task. However, there was no significant interaction between executive function and noise.

### Cognitive test battery

Table 1 shows the mean performance and standard deviation in the cognitive test battery. In the reading span task, the mean performance on semantic judgment was 50.46 (SD = 3.20) out of 54 possible responses, demonstrating that the participants performed that part of the dual task in accordance with instructions.

The correlations between the tests in the cognitive test battery are shown in Table 2. The overall pattern of significant correlations without correction for multiple comparisons is shown.

**Table 1:** **Mean performance in the cognitive test battery**.

**Cognitive Test**	**Mean**	**Standard deviation**
Reading span (total items correctly recalled)	29.7 (of 54 possible)	6.75
Letter memory test (recall of last four letters)	41.7 (of 48 possible)	4.6
Simon task (RT ms difference between incongruent and congruent trials)	83.74	55.23
TRT (Percentage unmasked text)	46.84	3.95
Delayed recall of reading span test (correctly recalled words)	21.7	16.33

**Table 2:** **Coefficients of correlations (Pearson’s *r*) between age and cognitive test scores**.

	**Age**	**Reading span**	**Letter memory test**	**Simon task**	**TRT**	**Delayed recall of reading span test**
Age		−0.05	0.42	−0.36	0.04	−0.16
Reading span			0.60**	0.07	−0.55*	0.75**
Letter memory test				−0.01	−0.55*	0.01
Simon task					0.30	0.20
TRT						−0.15

* Correlation is significant at the 0.05 level (2-tailed)

** Correlation is significant at the 0.01 level (2-tailed)

The performance in the reading span test was significantly associated with performance in the letter memory test and the delayed recall of the reading span test. The performance in the TRT was significantly associated with the performance in the reading span and letter memory tests (c.f. Besser et al., 2013).

Table 3 shows the correlations between cognitive tests and the SNR rendering 84% speech intelligibility for SSSW noise and actual speech intelligibility at estimated 90% intelligibility for SSSW and ISTS noise. The speech intelligibility scores in ISTS noise were associated with performance in the Simon task and TRT. Since the TRT score is the average percentage of unmasked text, a higher score in TRT indicates poorer performance. Similarly, in the Simon task, the score is the difference in reaction time between the incongruent and the congruent condition, so a greater difference indicates poorer inhibition ability.

**Table 3:** **Coefficients of correlations (Pearson’s *r*) between cognitive tests and speech intelligibility**.

	**SNR at 84% speech intelligibility**	**Actual speech intelligibility at estimated 90% level in SSSW noise**	**Actual speech intelligibility at estimated 90% level in ISTS noise**
Reading span	0.14	−0.13	0.14
Letter memory test	0.07	0.01	0.29
Simon task	−0.11	−0.19	−0.57**
TRT	−0.38	−0.27	−0.46*
Delayed recall of reading span test	−0.11	−0.36	−0.09

* Correlation is significant at the 0.05 level (2-tailed)

** Correlation is significant at the 0.01 level (2-tailed)

The correlations between CSCT and the battery of cognitive tests are shown in Table 4. To gain a detailed picture of the association between CSCT and cognitive function, we looked at the CSCT overall as well as factor-wise. Performance on the letter memory test was associated with CSCT irrespective of how scores were split, except when CSCT was performed in SSSW noise. There was no correlation between Simon and CSCT performance. However, TRT was associated with CSCT performance in inhibition conditions. TRT was also associated with performance in A-only modality and high load conditions of CSCT. Performance in reading span was associated with CSCT performance in quiet. Delayed recall of reading span test performance was not associated with performance in CSCT in any of the conditions. The correlation between overall performance in CSCT and TRT tended towards significance (*r* (20) = −0.43, *p* = 0.06).

**Table 4:** **Coefficients of correlations (Pearson’s *r*) between collapsed CSCT and cognitive test scores**.

**CSCT**	**Reading span**	**Simon task**	**Letter memory test**	**TRT**	**Delayed recall of reading span test**
Overall	0.25	−0.18	0.67**	−0.43	−0.08
Updating	−0.01	−0.24	0.44*	−0.11	−0.15
Inhibition	0.41	−0.06	0.65**	−0.58**	0.12
AV	0.24	−0.18	0.53*	−0.27	0.03
A-only	0.21	−0.14	0.70**	−0.51*	−0.05
Low load	−0.04	−0.23	0.57**	−0.24	−0.32
High load	0.35	−0.12	0.60**	−0.46*	0.15
Quiet	0.44*	0.15	0.73**	−0.37	0.12
SSSW	0.06	−0.20	0.30	−0.24	−0.11
ISTS	0.21	−0.31	0.57**	−0.40	−0.01

* Correlation is significant at the 0.05 level (2-tailed)

** Correlation is significant at the 0.01 level (2-tailed)

## Discussion

In the present study, CSC was investigated in young adults with normal hearing. The CSCT was administered in quiet as well as in steady state (SSSW) and speech-like (ISTS) noise at individual speech intelligibility levels approximating 90%. CSCT scores were higher in inhibition conditions compared to updating conditions and when memory load was low compared to when it was high, in line with previous findings (Mishra et al., 2013). SSSW noise reduced CSCT performance as predicted and seeing the talker’s face counteracted the noise decrement. However, ISTS noise did not reduce CSCT performance. In quiet conditions, visual cues reduced performance as predicted.

There was no interaction between executive function and noise; hence our prediction that the scores in the inhibition subset of CSCT would be reduced more than in the updating subset in modulated noise was not supported. There was no overall association between CSCT performance and WMC but updating capacity predicted CSCT performance in all conditions.

### CSCT performance in quiet

As predicted, we found higher CSCT scores in A-only modality compared to AV modality in quiet conditions. It has been argued that integration of visual information from the face of a talker with the speech produced by that talker does not consume cognitive resources (Campbell, 2009; Baddeley, 2012; Moradi et al., 2013; Yovel and Belin, 2013). However, it seems that when speech has to be processed executively, superfluous information carried in the visual stream may reduce performance (Fraser et al., 2010; Gosselin and Gagné, 2011; Mishra et al., 2013). This may be because executive load makes it difficult to prioritize task-related processing in the presence of low priority stimuli (Lavie, 2005). In the quiet conditions of the CSCT, the auditory cues alone provide young adults with normal hearing thresholds with sufficient information for performing the executive tasks. Thus, for these individuals, the visual cues may simply compete for cognitive resources without enhancing task performance.

### Effect of noise and modality of presentation on CSCT performance

As predicted, CSCT scores were lower in noise than in quiet. However, this applied only to performance in SSSW. Based on the findings of Ng et al. (2013), we expected the CSCT scores to be lower in speech-like noise compared to steady state noise. However, contrary to our expectation, we found that CSCT scores in ISTS noise was significantly higher than in SSSW noise, despite the fact that there was no difference in either SNR or speech intelligibility between the two noise conditions. Also, CSCT performance in ISTS noise did not differ significantly from performance in quiet even in the A-only modality, although it is conceivable that potential differences in CSCT performance in ISTS noise and quiet were concealed by near-ceiling performance. Preserved CSCT performance in ISTS noise may be explained by a selective attention mechanism that comes into play when speech stimuli are presented against a background of speech-like noise (Zion Golumbic et al., 2013); the target speech stimuli are dynamically tracked in the brain but interfering noise is not tracked. This finding suggests that selective attention at higher cortical levels suppresses interfering speech-like noise at the perceptual level and may provide richer representation of the target speech stimuli in memory. In SSSW noise, it is likely that selective attention to the speech stimuli could not be achieved due to the lack of modulation in the interfering noise resulting in a failure to segregate the speech stimuli from the SSSW noise (c.f. Helfer and Freyman, 2005). Pichora-Fuller et al. (1995) have argued that although speech information has been perceived, it may not be adequately encoded in memory for retrieval. In this study, the speech intelligibility performance in SSSW and ISTS noise was similar, but the representation of the target speech stimuli in memory may have been impoverished in SSSW noise due to lack of segregation from noise, but not in ISTS noise. This may have led to lower CSCT scores in SSSW noise than in quiet and ISTS noise.

We also predicted that seeing the face of the talker while performing the CSCT would counteract the negative effects of noise by providing a richer representation of the to-be-remembered items and this is what we found in SSSW noise. Indeed, there was no significant difference in performance in the AV modality between any of the noise conditions including quiet. These findings demonstrate that when noise disrupts executive processing of speech, seeing the face of the talker counteracts the disruptive effect of the noise.

Significant correlations between performance on the Simon task and TRT and speech intelligibility in ISTS demonstrate that listening in ISTS noise is cognitively demanding. The correlation between speech performance in ISTS and performance in the Simon task suggests that inhibition skills may come to the fore to suppress irrelevant information during memory encoding (Janse, 2012) and the association with TRT suggests a role for linguistic closure during speech perception performance in noise when irrelevant cues have to be disregarded (Zekveld et al., 2012, 2013; Besser et al., 2013). However, these demands on cognitive resources while listening in ISTS do not seem to influence CSC as CSCT scores were higher in ISTS compared to SSSW noise. Our interpretation is that while cognitive resources relating to inhibition and linguistic closure are employed for perceiving speech in presence of noise, other higher cognitive processes selectively attenuate the interfering modulated noise such that richer representation of target items in working memory can be achieved. This leads to less load on CSC. This effect may be similar to the benefit afforded by visual cues in terms of generating a richer representation of the items. In the present study, we do not find a significant difference between the performance in SSSW noise in AV modality and ISTS noise in A-only modality. This finding suggests that for young adults with normal hearing, selective attention to speech in the presence of speech-like noise enriches the representation of the target stimuli in working memory in much the same way as the presence of visual cues.

### Correlations between CSCT and the cognitive test battery

#### Working memory

We have previously reported evidence that CSC is not related to WMC measured using the reading span test (Mishra et al., 2013). The reading span test is a well-established test of verbal WMC (Daneman and Carpenter, 1980; Rönnberg et al., 1989) that has proved to be a potent predictor of the ability to unravel linguistic complexity (Unsworth et al., 2009) and in particular the ability to understand speech under adverse conditions (Akeroyd, 2008; Besser et al., 2013). In particular, it has proved useful as a way of probing the simultaneous storage and processing capacity that characterizes WMC, through the unimpaired modality of vision in persons with hearing impairment (Classon et al., 2013; Ng et al., 2013). Thus, we consider it to be the most suitable measure of general WMC. Although the concept of CSC is related to WMC in the sense that both involve temporary maintenance and processing of information, it is not necessarily the case that CSC can be assessed by simply measuring WMC. This is because general WMC may be depleted in different ways under different sets of listening conditions and reduced ability to deploy one set of processing skills, such as inhibition, may be compensated for fully or partially by other skills, such as updating. Thus, we did not expect CSCT performance to correlate with reading span performance, our measure of WMC in the present study. Indeed, there was no overall correlation, but CSCT performance in quiet conditions did correlate positively and significantly with WMC although not in either of the two noise conditions. On the face of it, it would appear that the quiet conditions in the present study are those that most closely resemble the conditions in the previous study (Mishra et al., 2013). However, there were several methodological differences in the present study compared to Mishra et al. (2013) that may help explain the difference in the pattern of correlations between the two studies. Button-press responses were used in Mishra et al. (2013), whereas voice responses were used in the present study. Button-press responses are more cognitively demanding than vocal responses as they require the synchronization of motor response and visual scanning of buttons. Thus, in our previous study (Mishra et al., 2013) general cognitive resources were probably being engaged for motor planning during the response phase of the task, reducing CSC and leading to a lack of correlation with reading span performance. In the present study, however, CSC in quiet conditions is likely to be more similar to independently measured WMC, which may explain the intercorrelation. Notwithstanding, adjustment for multiple comparisons renders this isolated correlation insignificant.

#### Executive functions

It is notable that performance in none of the tasks in the cognitive test battery predicted CSCT performance in SSSW noise. This may be because the cognitive skills measured by the test battery in the present study do not contribute towards executive processing of items presented in SSSW noise. However, letter memory performance predicted performance in both quiet and ISTS conditions. In quiet, there is no interfering noise to disrupt representation of the target stimuli in working memory. We have argued that in ISTS noise, good representation in working memory can be achieved by selective attention to speech (Zion Golumbic et al., 2013) whereas this is not the case in the presence of SSSW noise. The association of letter memory performance and CSCT performance in quiet and ISTS noise suggests that updating skills play an important role during processing of encoded representations. This in turn suggests that CSC in young persons with normal hearing thresholds may capitalize on updating ability when cognitive representations are rich.

Because the CSCT taps executive functions, we expected CSCT performance to correlate with the independent tests of executive function (c.f. Mishra et al., 2013). In particular, we expected updating ability to facilitate CSCT performance, especially in updating conditions, and inhibitory ability to facilitate CSCT performance in inhibition conditions. Performance on the letter memory task (Miyake et al., 2000) which was our independent test of updating correlated positively and significantly with performance in both the updating and inhibition conditions of the CSCT. Performance on the Simon task (Simon, 1969; Pratte et al., 2010) which was our independent test of inhibition skill did not correlate with CSCT under any conditions of the CSCT. Performance on letter memory and Simon tasks was not significantly related. In the present study, target items were presented in two different kinds of background noise for two out of three lists in an unpredictable manner. This means that the participants were probably always on the alert, at least at the beginning of each list, to cope with background noise, even when lists were presented in quiet. In other words, cognitive resources, probably inhibition skills, were probably always allocated, even when not specifically needed. This may have meant that participants had fewer inhibition resources available to engage in executive processing of the numbers. We have argued that a cognitive resource that is depleted in the act of speech perception may be compensated for fully or partially by another cognitive function during further processing. The pattern of correlations between CSCT and the cognitive test battery suggests that consistent demands were made on updating skills while inhibition skills had less impact. The explanation may be that during executive processing of numbers, updating skills compensated for the unavailability of inhibition skills engaged in preparing for noise.

Inhibition skills were significantly related to the intelligibility of the stimuli in ISTS noise, which suggests that inhibition skills were required to suppress the irrelevant information present in the ISTS noise during speech perception although they did not enhance memory performance or influence CSC. This may be because the inhibition resources were reduced during perception of numbers in noise and other cognitive skills like linguistic closure and updating were used to perform the inhibition task of CSCT.

#### Linguistic closure

The association of TRT with the inhibition subset of CSCT suggests that the ability to make use of linguistic closure is related to the processing required to identify and keep in mind auditory two-digit numbers of a certain parity and voice. The common factor may be an underlying ability to generate a coherent response on the basis of diverse pieces of information. The main effects of memory load and modality in CSCT performance revealed that the CSCT scores were lower when memory load was high and when visual cues were absent, suggesting that under these conditions the CSCT task is more difficult. The fact that performance in TRT was associated with CSCT performance in these particular conditions suggests that linguistic closure ability enhances CSCT performance when the task is difficult. This interpretation is supported by evidence that TRT predicts recall of speech heard in noise together with irrelevant visual cues (Zekveld et al., 2012). However, when visual information was available, CSCT performance in the present study was not associated with the TRT performance. This is in line with our prediction that AV integration is not cognitively taxing (Baddeley, 2012; Moradi et al., 2013). Further, we predicted that overall CSCT performance would be associated with performance in TRT and a tendency towards this association was found suggesting that overall performance in CSCT is predicted by an ability to make use of linguistic closure.

#### Episodic long-term memory

Finally, the delayed recall of the reading span stimuli measuring episodic LTM was not associated with performance in CSCT. Thus, no support was found for an association between episodic LTM and CSC in young adults with normal hearing. Recent work shows that hearing loss is associated with decline in LTM and it has been suggested that the mechanism behind this association is that hearing loss leads to more mismatch due to poor audibility and distortion of the input signal and thus less access to LTM (Rönnberg et al., 2011). Hence, it can be expected that an efficient LTM may facilitate processing of speech in adverse conditions, even though no such evidence was found for the participants with normal hearing in the present study.

## Cognitive spare capacity

Cognitive resources are consumed in the act of listening (Pichora-Fuller, 2009; Rönnberg et al., 2013). Individual WMC capacity is associated with the ability to recognize speech in noise and the reading span task has proved to be a particularly potent predictor (Akeroyd, 2008; Besser et al., 2013). However, because available cognitive resources may be deployed differently under different listening conditions (Pichora-Fuller and Singh, 2006; Pichora-Fuller, 2007), it is important to gain an understanding of CSC which is the cognitive reserve that has been depleted by listening under adverse conditions. This requires an experimental approach. The results of the present study show that the CSCT may be a useful tool in this enterprise. They provide a baseline performance level for CSCT in quiet and in noise for young adults with normal hearing. The next step is to investigate CSCT performance in persons with hearing impairment. In the future, we aim to develop a simplified version of the CSCT which can be used for evaluation of hearing aid fitting and different signal processing strategies used in hearing aids. By using CSCT we will be able to show the influence of signal processing on memory for heard speech. We believe that CSCT performance can provide us with a snap-shot of how hearing-aid signal processing influences cognitive demands in communicative situations (Rudner and Lunner, 2013).

## Conclusion

 The results of the present study replicate the results of Mishra et al. (2013) by showing that CSC in young adults with normal hearing thresholds is sensitive to storage load and executive function but not generally related to WMC and that availability of visual cues may hinder executive processing of speech heard in quiet. They also extend these results by showing that even when speech intelligibility is high, steady-state noise may lower CSCT performance but that this decrement can be restored when the talker’s face is visible, probably by aiding segregation of target items and thus enriching their cognitive representation. Speech-like noise did not reduce CSCT performance, which was contrary to our prediction. We suggest that selective attention was used to ignore the speech-like background noise to provide an enriched representation of target items similar to that obtained in quiet. The overall pattern of results suggests that updating skills play a key role in exploiting CSC and may provide high-level compensation when inhibition skills are engaged in low-level processing.

## Conflict of interest statement

The authors declare that the research was conducted in the absence of any commercial or financial relationships that could be construed as a potential conflict of interest.
